# Site Injection Reaction, Maculopapular Rash, and Rosacea Exacerbation after COVID-19 Vaccination

**DOI:** 10.1155/2022/9944727

**Published:** 2022-04-14

**Authors:** Younes Benyamna, Farah Marraha, Ibtissam Al Faker, Hanane Chahoub, Najlae Rahmani, Yasmine Rkiek, Soukayna Kabbou, Driss Soussi Tanani, Salim Gallouj

**Affiliations:** ^1^Department of Dermatology, University Hospital Center of Tangier, Tetouan, Al Hoceima, Morocco; ^2^Faculty of Medicine and Pharmacy Tangier, Abdelmalek Essaadi University, Tangier, Morocco; ^3^Department of Pharmacology and Pharmacovigilance, University Hospital Center of Tangier, Tetouan, Al Hoceima, Morocco

## Abstract

To date, the occurrence of adverse events following immunization after COVID-19 vaccine is rare, and their report is still very poor; however, causality assessment is conducted to identify the associated cause, if they occur. In this case report, we present a case of an association of three cutaneous manifestations (maculopapular exanthem with enanthem, site injection reaction, and rosacea exacerbation) occurring three days after taking the first dose of AstraZeneca AZD1222 vaccine.

## 1. Introduction

In November 2019, the first cases of novel beta-corona virus SARS-CoV-2 were detected [1].

The virus has since spread globally at an alarming rate, and a SARS-CoV-2 global pandemic was declared by the World Health Organization (WHO) in March 2020 [2].

Beside its high spread rate, SARS-CoV-2 was shown to be responsible of severe acute respiratory distress syndrome and death. Thus, there was a race against time to find a solution that may slow down the coronavirus disease 2019 (COVID-19). Many pharmaceutic and nonpharmaceutic options were used [3], but only a vaccine seems efficient to stop or, at least, slow down the spreading of the virus by promoting herd immunity through massive vaccination campaigns.

Traditionally, vaccine development takes more than 10  years, but the COVID-19 pandemic has demonstrated the urgency for an unprecedented quick vaccine development. A safe and efficacious vaccine had to be developed in less than 6–18 months.

According to the WHO, the production of a vaccine passes conventionally through different stages. First, screenings and evaluations are necessary to determine which antigen should be used. The retained vaccine is then tested in animals. This is called the preclinical stage. If the vaccine triggers an immune response, it is then tested in human clinical trials in three phases. Safety of the vaccine is generally assessed in the clinical trials. Once the clinical trials are achieved, only approval is left. An authorized vaccine is now ready to be introduced, but a further monitoring is necessary; pharmacovigilance takes place [4].

Pharmacovigilance has been defined by the WHO as the “science and activities relating to the detection, assessment, understanding and prevention of adverse effects or any other possible drug-related problems” [5].

In Morocco, the vaccination is provided using either the inactivated vaccine Sinopharm BBIBP-CorV or the AstraZeneca AZD1222 vaccine using adenoviral vectors. The two vaccines present overall a good safety [6, 7].

Since the first administrated doses, the clinicians focus was on the surveillance of possible adverse events due to vaccine.

In this article, we report a rare case of an association of three cutaneous reactions after a vaccination with the AZD1222 vaccine.

## 2. Case Presentation

A 21-year-old female patient with a medical history of iron deficiency anemia on oral iron supplementation consulted for generalized maculopapular exanthem with enanthem.

A careful history taking was established. In fact, the patient has beneficiated from COVID-19 vaccination, 5 days before consultation. The onset was marked 3 days after vaccination by redness with itching in the site of injection (left arm). The erythema quickly became generalized on the 3rd day, producing a maculopapular exanthem. Furthermore, our patient did not show any general signs or pruritus (except of the site of the injection). Of note, the patient does not report any allergic history.

**Figure 1:**
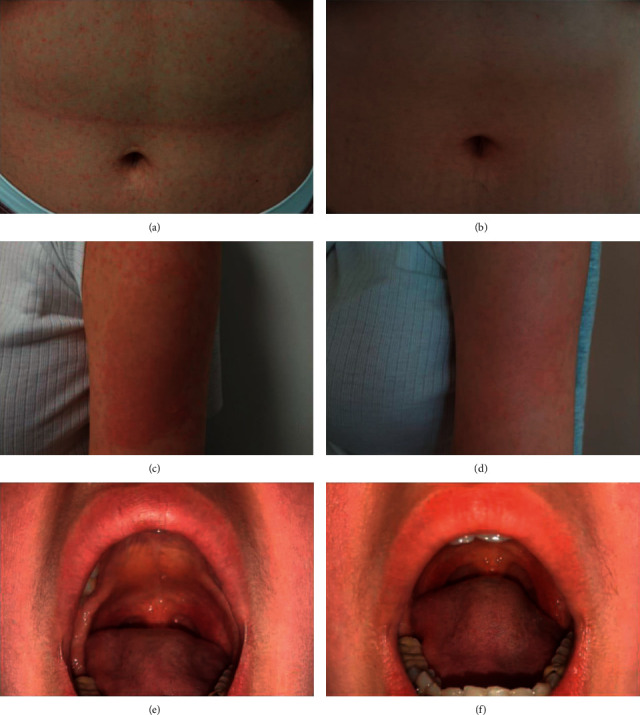
Clinical presentation. (a) The maculopapular exanthem at admission. (b) Regression of the maculopapular exanthem after a week. (c) Erythema of the injection site at admission. (d) Injection site after a week. (e) The enanthem at admission. (f) Persistence of enanthem after a week.

**Figure 2:**
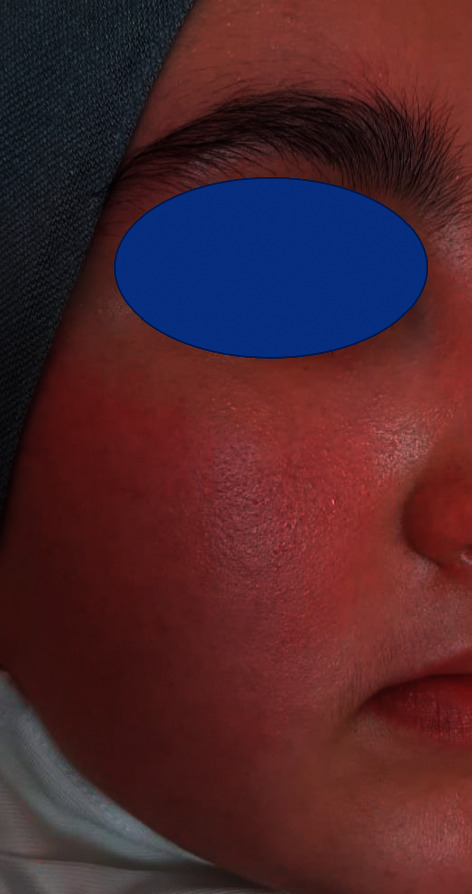
Face erythema.

On the clinical examination, 5 days after vaccination, there was a generalized skin eruption consisting of macules and papules which do not form a scale (maculopapular exanthem), involving the trunk and limbs and sparing the palmoplantar regions (Figure 1). On the face, the patient also presented erythema which was in favor of a rosacea (after clinical and dermoscopic examination) (Figure 2). At the injection site, the patient presented an erythematous patch (8 × 5 cm of diameter). In addition to that, enanthem was found at the mucosal examination (Figure 1).

There was no associated lymphadenopathy, and other systemic examinations were normal. Laboratory investigations revealed normal leucocyte count (5.76 × 103/*μ*L) with a slightly elevated granulocytes percentage (72.6%) and normal lymphocytes and eosinophils percentage (respectively, 22% and 0.2%). C-reactive protein, liver function tests, and renal profile were normal (CRP = 2.4 mg/l, ALT = 10.4 U/l, AST 15 U/l, urea = 0.14 g/l, and creatinine = 5.1 mg/l).

As it is invasive, skin biopsy has not been done.

The patient was started on H1 antihistamine pills and topical corticosteroids with emollients. For her rosacea, the patient was put under topic metronidazole. The MPE lesions and site injection erythema improved remarkably after 5 days of treatment, but the enanthem persisted and further follow-up was scheduled to monitor her rosacea.

## 3. Discussion

Just like any approved medicine, any approved vaccine carries some risk of side effects.

The cutaneous adverse effects of vaccines include local and generalized reactions. Some mechanisms of action are known, and others are not well elucidated yet. The main cause of those reactions is allergic or pseudoallergic. The hypersensitivity can be immediate-type or delayed-type, and it is due to the vaccine or one of its components [8].

As the size of the populations studied in the premarketing phase is limited and the period of observation is short (especially with the COVID-19 vaccine, which was produced in less than one year), safety data are usually very limited; then, the side effects reported to each vaccine are very limited. Some rare or severe reactions may be reported only after commercialization of the vaccine. Generally, postmarketing surveillance of vaccines relies mainly on the spontaneous reporting system (SRS) (which is the case for our patient). This method is crucial to generate alerts, but pharmacoepidemiology studies are necessary to confirm the alerts identified by spontaneous reporting. This is the huge importance of vaccines pharmacovigilance [9].

In our case, while the imputability of the skin manifestations observed to the administered vaccine is high (considering the onset and the evolution), scientific proof of a cause and effect relationship is very difficult to obtain.

Our patient presented three different adverse effects in which the severity risk was qualified as mild: injection site reaction, maculopapular exanthem (MPE) with enanthem, and rosacea exacerbation.

According to literature, injection-site reactions are the most common form of reaction that follows an injectable vaccine administration [10].

Clinical presentation includes erythema and/or swelling and/or tenderness. Generally, this reaction resolves spontaneously within a few days to weeks. However, local care can be taken to reduce the symptoms and accelerate healing. Of note, site injection reactions may occur with any vaccine and are not specifically related to any of the vaccine components [8].

What about the maculopapular exanthem? To answer this question, it would be interesting to take a look at the composition of the vaccine taken by our patient. In fact, AZD1222 vaccine consists of a replication-deficient chimpanzee adenoviral vector ChAdOx1, containing the SARS-COVID-19 structural surface glycoprotein antigen (spike protein; COVID-19) gene [6].

In addition to the principal fraction of this vaccine, AZD1222 contains some excipients: L-histidine, L-histidine hydrochloride monohydrate, magnesium chloride hexahydrate, polysorbate 80, ethanol, sucrose, sodium chloride, disodium edetate dihydrate, and water for injection [11]. Among all these excipients, the most interesting component to highlight is polysorbate 80. It is used as a synthetic nonionic surfactant commonly used in vaccines and drugs as a solubilizer, stabilizer, or emulsifier and also used to prevent protein adsorption and/or aggregation. In fact, it has been noted that polysorbate 80 is a biologically and possibly pharmacologically active compound and consequently may alter the pharmacologic properties of the drug it is formulated with or may itself directly mediate adverse events. Consequently, polysorbate 80 has been implicated in some of the adverse reactions associated with drugs formulated with this vehicle [12].

Its properties reside on its capacity of activating the complement system and then may lead to phagocytosis, stimulation, and recruitment of white blood cells or perforation of cell membranes. We believe that polysorbate 80 leads to immunologic side effects such as acute hypersensitivity and systemic immune reactions [13]. This explanation seems recomforting. Yet, in our case, the rash occurred 3 days after vaccination, which cannot be taken as an immediate hypersensitivity reaction.

Maculopapular rashes are described as a rare adverse event that may occur after immunization. It is also described as a delayed-type hypersensitivity reaction. In fact, the pathogenesis of this reaction is not fully understood. However, basophils activation and a reaction to circulating immune complexes take a major role in the pathogenesis [14].

Furthermore, giving that the commonest etiologies of a MPE are drugs and viral infections and that the vaccine taken by the patient is an adenovirus-vectored virus, we cannot discard a possible infection to adenovirus. Indeed, as described in literature, exposure to viruses may occur at mucosal surfaces or abraded skin sites and may or may not be pruritic. Apropos, this rash is not directly due to viral replication, but a hypersensitivity reaction to the virus.

Niedermeier et al. [15] reported a maculopapular rash presented in a 6-year-old patient who presented, few days before, an upper respiratory tract infection to adenovirus. This eruption resolved spontaneously within 4 weeks.

In addition to the two previous presentations, our patient presented a facial erythema. After the clinical and dermoscopic examination, we retained rosacea as diagnosis.

Rosacea is a chronic inflammatory dermatological condition that concern the central facial skin [16].

It has rarely been described as an adverse effect of treatment or vaccination. However, some cases of rosacea associated with nicotinic acid (niacin, vitamin B3), high doses of vitamin B6 and B12, or etanercept have been reported in literature [16, 17]. On the other hand, several factors are well known to initiate or aggravate rosacea. These factors may be endogenous or exogenous, and they include ultraviolet (UV) radiation, heat, cold, stress, spicy food, and microbes [18].

The mechanisms in which those factors work are not well known. Yet, the role of immunity has been described.

Adaptive and innate immune systems might take a major role in the rosacea pathophysiology. Furthermore, several activation pathways have also been described, such as inflammasome (NALP3), TLR2, and TRPV1. These pathways are triggered in response to several factors previously mentioned.

For our patient, there is no clear cause and effect relationship between vaccination and rosacea. In contrast, emotional stress is described to trigger the three activation pathways mentioned above [19]. Furthermore, the body response to vaccine confers an adequate environment to rosacea, as both innate and adaptive immune systems are activated.

## 4. Conclusion

The postmarketing phase is crucial for monitoring possible adverse effects that may be related to new vaccines. In this case, we report three cutaneous adverse effects (site injection reaction, maculopapular rash, and rosacea exacerbation) observed in the same patient after taking her first dose of AstraZeneca AZD1222 vaccine. The imputability is high; however, further research studies are needed to confirm the causal link with this vaccine.
